# Determinants of tobacco use by students

**DOI:** 10.1590/S1518-8787.2017051006283

**Published:** 2017-04-24

**Authors:** Lorena Silva Vargas, Roselma Lucchese, Andrécia Cósmem da Silva, Rafael Alves Guimarães, Ivânia Vera, Paulo Alexandre de Castro

**Affiliations:** I Programa Municipal de Controle do Tabagismo. Secretaria Municipal de Saúde de Catalão. Catalão, GO, Brasil; II Departamento de Enfermagem. Universidade Federal de Goiás. Catalão, GO, Brasil; III Universidade Estadual de Goiás. Ipameri, GO, Brasil; IV Faculdade de Enfermagem. Universidade Federal de Goiás. Goiânia, GO, Brasil; V Departamento de Física. Universidade Federal de Goiás. Catalão, GO, Brasil

**Keywords:** Adolescent, Young Adult, Smoking, epidemiology, Family Relations, Risk Factors, Socioeconomic Factors

## Abstract

**OBJECTIVE:**

Estimate the prevalence and determinants of tobacco use by students.

**METHODS:**

This cross-sectional study, carried out between 2013 and 2014, evaluates 701 public school students between 10 and 79 years of age living in a city in the countryside of the State of Goias, Midwest of Brazil. A structured questionnaire collected the data and the predictor variables were demographic data, family nucleus, religion, physical activity practice, family functionality and parental smoking. Two multivariable models were implemented, the first for occasional tobacco use and the second for regular use, acquiring the measure of prevalence ratio (PR) and their respective 95%CI. Variables with p < 0.10 were included in Poisson regression models with robust variance to obtain the adjusted PR (adPR) and 95%CI. The Wald Chi-Squared test examined the differences between proportions, and values with p < 0.05 were statistically significant.

**RESULTS:**

The prevalence of occasional and regular tobacco use were 33.4% (95%CI 29.8–36.9) and 6.7% (95%CI 5.0–8.8), respectively. The factors associated with occasional tobacco consumption were age of 15 to 17 years (adPR = 1.98) and above 18 years (adPR = 3.87), male gender (adPR = 1.23), moderate family dysfunction (adPR = 1.30), high family dysfunction (adPR = 1.97) and parental smoking (adPR = 1.60). In regards to regular consumption of tobacco, age above 18 years (adPR = 4.63), lack of religion (adPR = 2.08), high family dysfunction (adPR = 2.35) and parental smoking (adPR = 2.89) remained associated.

**CONCLUSIONS:**

Students exhibit a high prevalence of occasional and regular tobacco use. This consumption relates to sociodemographic variables and family dysfunction.

## RESUMO

**OBJETIVO:**

Estimar a prevalência e determinantes do consumo de tabaco por estudantes.

**MÉTODOS:**

Neste estudo de corte transversal, realizado entre 2013 e 2014, avaliamos 701 estudantes entre 10 e 79 anos de escolas públicas de um município do interior do estado de Goiás, Centro-Oeste do Brasil. Os dados foram coletados por meio de um questionário estruturado, cujas variáveis preditoras foram: dados sociodemográficos, núcleo familiar, religião, prática de atividade física, funcionalidade familiar e pai ou mãe fumante. Foram implementados dois modelos multivariáveis, o primeiro para uso de tabaco na vida e o segundo para uso regular, obtendo-se como medida de efeito razão de prevalência (RP) e seus respectivos IC95%. Variáveis com p < 0,10 foram incluídas em modelos de regressão de Poisson com variância robusta, para obtenção da RP ajustada (RPaj) e IC95%. O teste Qui-quadrado de Wald foi utilizado para testar as diferenças entre as proporções, e valores com p < 0,05 foram considerados estatisticamente significantes.

**RESULTADOS:**

As prevalências de consumo de tabaco na vida e regular foram de 33,4% (IC95% 29,8–36,9) e 6,7% (IC95% 5,0–8,8), respectivamente. Os fatores associados ao consumo de tabaco na vida foram: idade de 15 a 17 anos (RPaj = 1,98) e superior a 18 anos (RPaj = 3,87), sexo masculino (RPaj = 1,23), moderada disfuncionalidade familiar (RPaj = 1,30), elevada disfuncionalidade familiar (RPaj = 1,97) e pai ou mãe fumante (RPaj = 1,60). Em relação ao consumo regular de tabaco, permaneceram associados a idade superior a 18 anos (RPaj = 4,63), não possuir religião (RPaj = 2,08), elevada disfuncionalidade familiar (RPaj = 2,35) e pai ou mãe fumante (RPaj = 2,89).

**CONCLUSÕES:**

Estudantes apresentam elevada prevalência de consumo de tabaco na vida e regular. Esse consumo associa-se com variáveis sociodemográficas e disfuncionalidade familiar.

## INTRODUCTION

Smoking, a huge public health problem throughout the world, is responsible for the death of 6 million people every year, mostly through chronic noncommunicable diseases, such as neoplasms, respiratory and cardiovascular diseases^a,b^. Use of tobacco causes an estimated 71% of deaths by lung neoplasms, 42% of chronic respiratory diseases and 10% of cardiovascular diseases. In 2012, the World Health Organization (WHO) estimated a 36.1% and 6.8% prevalence of tobacco use in men and women over 15 years of age, respectively^c^.In Brazil, the rate is 21% of men and 12% of women^c^.

Adolescents and young adults, especially students, correspond to groups with high vulnerability to tobacco consumption. In fact, the use of tobacco, especially when accessed by electronic devices (e-cigarettes), has increased in these populations^1^. In developed countries like the United States (US) and Canada, the frequency of tobacco use in the last 30 days is 12.7% and 14.4% in students, respectively. In developing countries, these rates reach 9.2% in Uruguay, 12.5% in Colombia, 12.3% in Bolivia, 14.7% in Paraguay, and 18.7% in Argentina^4^. In Brazil, a study conducted with 61,037 students of 26 capital cities and the Federal District, National Survey of School Health (PeNSE) participants, estimated the prevalence of occasional tobacco use and in the last 30 days of 22.7% and 7.1%, respectively^2^.

Multiple factors are associated with tobacco use in these populations, such as sociodemographic characteristics (age, gender, religion)^3^; factors related to the school environment (friend’s influence, friends who smoke, school disapproval)^4^; and behavioral factors (illicit drug use, early sexual activity, among others)^5^. A systematic review looking to analyze tobacco use in adolescents and identify subgroups at greatest risk found a 9.3% average prevalence of tobacco use in the last 30 days in this population. The authors identified the following variables as main risk factors: increasing age, use of other substances, suicide ideation, lack of religion and parental exposure to smoking products^3^.

Despite numerous studies investigating the risk factors of tobacco use, few investigations have focused on family influence in the use of that substance, and on family dysfunction in particular. Research indicates that the family environment and conflictual family relationships are directly associated with an increase in the odds of experimentation, regular consumption of tobacco and nicotine addiction in students. Factors such as the influence of smoker parents and siblings, having single or divorced parents, low education level, lack of family support and participation, and no monitoring and limitations by guardians are responsible for increased use in adolescents and young adults^6,7^.

Studies indicate high smoking prevalence in students^4-6^. Hence, to plan public policies and intervention strategies for prevention and control tobacco use damages, it is necessary to investigate the school environment and the family relationships of students, as well as reviewing previously studied variables and the influence of family functionality in the epidemiology of tobacco use from consistent and validated instruments on family dynamics. Following this perspective, this study’s objectives were to estimate the prevalence and determinants of tobacco use by students.

## METHODS

This is a cross-sectional study carried out with students from urban and rural public schools in a countryside city in Goiás. The data collection took place between November 2013 and February 2014. Subjects were 10 years old or older and regularly enrolled in schools of primary and secondary education or in the course for Youth and Adult Education (EJA). For this investigation, the cut-off age was 10 years, set by the World Health Organization (WHO) as the beginning of adolescence^d^.

For the sample calculation, we used a population of 7,700 students enrolled in 23 schools in the municipality, with 95% confidence level, 5% sampling error and 30% prevalence of regular tobacco consumption, found in research carried out in students of Goiânia, State of Goiás^e^, with a minimum size of 542 people. This number was increased by 20%, considering possible losses or refusals, totaling a sample of 651 subjects. Due to the availability of time and physical space in some schools, we interviewed over 50 individuals, ending the final sample with 701 students.

For participant selection, we used a probabilistic sampling. Of all municipal schools (n = 45), 23 had students within the age range of this study. School units were invited to participate in the research. One school unit refused to attend, claiming it would happen during the finals period. Thus, there was a redistribution of the number of subjects assigned to the schools (n = 7). Initially, there was a random selection of classes and, for every classroom, individuals were drawn and invited to participate as subjects in the research. Selected students under the age of 18 received a consent term (CT) for them and their parents to sign and an informed consent term (ICT) for their parents or guardians to sign. The choice of the number of students selected in each school was proportional to the number of students enrolled in each institution. Cases of non-submission of the TA and the ICT or refusal (students under the age of 18 years) accounted for 11% of the sample. Hence, a new individual was selected randomly, following the sampling standards of conduct. Mental health specialists and researchers developed the data collection instrument using variables tested and validated in other studies in populations of adolescents and young adults. There was also a questionnaire recommended by WHO for the evaluation of family functionality of adolescents and young adults^f^.

Tablets, palmtops, and laptops were used to collect data through a semi-structured questionnaire computerized in Google Docs^®^ , making the use of printed materials unnecessary. The information was automatically stored in spreadsheets. All participants were interviewed face-to-face in a private location on the educational institutions’ premises.

This study’s outcome variables were: “tobacco use at least once in life” and “regular consumption of tobacco”, defined by the use on one or more days in the past 30 days^2^. This study analyzes the following predictor variables: sociodemographic data (age, gender, children, and skin color), family nucleus (living with family or friends; alone), religion, physical activity practice, family functionality and parental smoking. The age variable categories were ≤ 14 years old, 15-17 years old and ≥ 18 years old. Factors associated with regular tobacco consumption were analyzed by grouping the ages as ≤ 18 years old and > 18 years old because there was no prevalence of tobacco use in adolescents aged 14 years old or less. Skin color was categorized as white or non-white (black, dark-skinned and yellow)^8^.

APGAR measured the family functionality. It is an instrument to assess a person’s satisfaction index with their family’s functioning according to five items, namely: Adaptation, Partnership, Growth, Affection, and Resolve. For each category, the family functionality received a score ranging from zero to two points. Thus, the sum of scores can range from zero to four, when there is high family dysfunction, from five to six, if moderate family dysfunction, and from seven to 10, the family’s functionality is good^f^.

The Fagerström test (FT) was applied to students who reported regular tobacco consumption. This tool is easy-to-use, can be self-applied, contains six items, and the sum of answers explains the degree of dependence on nicotine, with scores ranging from zero to ten points. For scores ranging from zero to two, the dependency is very low; three to four equals low dependence; five is medium dependence; six to seven equals high dependence, and eight to ten equals very high dependency^9^.

The family APGAR and FT instruments have undergone reliability testing with Cronbach’s alpha. The results were 0.70 for APGAR and 0.88 for FT.

The statistical program *Statistical Package for the Social Sciences* (SPSS), version 22.0, received data from the spreadsheets. Prevalence of occasional and regular tobacco use was calculated with 95% confidence intervals (95%CI). Two multivariable models were implemented to estimate the associated factors, the first for occasional tobacco use and the second for regular use, acquiring the measure of prevalence ratio (PR) and their respective 95%CI. Initially, there was a univariate analysis between the outcome variables and potential associated factors. Afterward, variables with p < 0.10 were included in Poisson regression models with robust variance to obtain the adjusted PR (adPR) and 95%CI. Age and gender were potential confounders and no variable was a modifier effect in the testing of collinearity. The final models were adjusted for all variables with p < 0.10 and potential confounders (age and gender). The Wald Chi-Squared test tested the differences between proportions, and values with p < 0.05 were statistically significant.

The Research Ethics Committee of the Universidade Federal de Goiás approved this study (Protocol 334.515/2013).

## RESULTS

Seven-hundred and one students participated in the study, 51.8% female and 48.2% male. The participant’s average age was 19.8 years old (SD = 9.9). As for skin color, approximately half (47.4%) self-declared as dark-skinned and the majority (87.2%) reported having a religion (Table 1).

**Table 1:** Sociodemographic profile of students from public schools in the countryside of Goiás, in the Midwest region of Brazil.

Variable	n*	%
Gender		
Female	363	51.8
Male	338	48.2
Age (years; average: 19.8; SD = 9.9)		
≤ 14	260	37.1
15–17	160	22.8
≥ 18	281	40.1
Skin color (self-declared)		
Yellow	36	5.1
White	236	33.7
Black	97	13.8
Dark-skinned	332	47.4
Religion		
Catholic	299	42.7
Evangelic	281	40.1
Spiritist	27	3.9
Other	4	0.5
None	90	12.8

* n = 701

The prevalence of occasional and regular tobacco use were 33.4% (95%CI 29.8–36.9) and 6.7% (95%CI 5.0–8.8), respectively. Table 2 presents the univariate analysis of potential factors associated with these usage patterns in the students studied.

**Table 2:** Univariate analysis of factors associated with occasional and regular tobacco consumption in students from public schools in the countryside of Goiás, in the Midwest region of Brazil.

Variable	Total	Lifetime tobacco consumption		Unadjusted PR		Regular tobacco consumption		Unadjusted PR	
				
	(n = 701)^a^	n	%	95%CI	p^b^	n	%	95%CI	P^b^
Age (years)									
≤ 14	260	33	12.7	1.00				-	-
15–17	160	43	26.9	2.11 (1.40–3.18)	0.000	10	6.2	1.00	
≥ 18	281	158	56.2	4.43 (3.16–6.19)	0.000	37	13.2	5.53 (2.79–10.94)	0.000
Gender									
Female	363	102	28.1	1.00		16	4.4	1.00	
Male	338	132	39.1	1.38 (1.12–1.71)	0.002	31	9.2	2.08 (1.15–3.73)	0.014
Children									
No	557	155	27.8	1.00		30	4.5	1.00	
Yes	144	79	54.9	1.97 (1.61–2.40)	0.000	17	11.8	2.19 (1.24–3.86)	0.007
Skin color									
White	236	75	31.8	1.00		17	7.2	1.00	
Non-white	465	159	34.2	1.07 (0.85–1.34)	0.525	30	6.5	0.89 (0.50–1.59)	0.707
Family nucleus									
Family	673	217	32.2	1.00		45	6.7	1.00	
Friends/Alone	28	17	60.7	1.88 (1.37–2.58)	0.000	7	7.1	1.06 (0.27–4.18)	0.925
Religion									
Yes	611	195	31.9	1.00		34	5.6	1.00	
No	90	39	43.3	1.35 (1.04–1.76)	0.023	13	14.4	2.59 (1.42–4.72)	0.002
Practices physical activity									
Yes	361	108	29.9	1.00		22	6.1	1.00	
No	340	126	37.1	1.23 (1.00–1.52)	0.046	25	7.4	1.20 (0.69–2.09)	0.506
Family APGAR									
Good functionality	537	28.5	28.5	1.00		26	4.8	1.00	
Moderate dysfunction	118	47	39.5	1.39 (1.07–1.81)	0.011	12	10.2	2.10 (1.09–4.04)	0.026
High dysfunction	46	34	73.9	2.59 (2.08–3.22)	0.000	9	19.2	4.04 (2.01–8.10)	0.000
Father or mother smokes									
No	271	55	20.3	1.00		6	2.2	1.00	
Yes	419	175	41.8	2.05 (1.58–2.67)	0.000	40	9.5	4.31 (1.85–10.00)	0.001

^a^ Number of valid responses.

^b^ Wald Chi-Squared test.

In the multivariate model, factors independently associated with occasional tobacco consumption were ages between 15 and 17 years old (adPR = 1.98) and above 18 years old (adPR = 3.87), male gender (adPR = 1.23), moderate family dysfunction (adPR = 1.30), high family dysfunction (adPR = 1.97) and parental smoking (adPR = 1.60). In regards to regular consumption of tobacco, age above 18 years (adPR = 4.63), lack of religion (adPR = 2.08), high family dysfunction (adPR = 2.35) and parental smoking (adPR = 3) remained associated factors in multivariate analysis.(Table 3)

**Table 3:** Factors associated with occasional and regular tobacco consumption in students from public schools in the countryside of Goiás, in the Midwest region of Brazil.

Variable	Adjusted PR	95%CI	p
Lifetime tobacco consumption^a^			
Age (15-17 years)	1.98	1.32–2.96	0.001
Age (> 18 years)	3.87	2.76–5.44	0.000
Male gender	1.23	1.01–1.50	0.032
APGAR (moderate dysfunction)	1.30	1.03–1.65	0.025
APGAR (high dysfunction)	1.97	1.48–2.63	0.000
Father or mother smokes	1.60	1.24–2.06	0.000
Regular tobacco consumption^b^			
Age (> 18 years)	4.63	2.31–9.27	0.000
Religion (no)	2.08	1.11–3.98	0.022
APGAR (high dysfunction)	2.35	1.10–5.02	0.026
Father or mother smokes	2.89	1.23–6.83	0.015

^a^ Model adjusted for age, gender, children, living arrangement, physical activity practice, family APGAR, and parental smoking.

^b^ Model adjusted for age, gender, children, religion, family APGAR, and parental smoking.

Of the subjects who reported regular tobacco use (n = 47), 74.5% (95%CI 60.5–85.0) presented nicotine dependence high/very hight in FT.

## DISCUSSION

The present study investigated the prevalence and determinants of tobacco use by students in a countryside city in Goiás. The results indicate a high prevalence of occasional and regular tobacco use, suggesting the students vulnerability for this behavior. Important variables were associated with tobacco consumption, such as increasing age, male gender, lack of religion and having a parent who smokes. As far as we know, this is the first study to assess the influence of family dysfunction and tobacco consumption in students using as an instrument recommended and validated by the WHO as an indicator. The findings indicate a strong association between family dysfunction and tobacco consumption in this population, which highlights the need for further research and knowledge production and evidence that subsidize the activities of health professionals as well as teachers, with a focus on family-centered interventions.

Results indicate high prevalence of occasional tobacco use (33.4%), similar to the rate found in students from developed countries, such as the USA (34.7%)^1^ and Canada (36.6%)^10^, and in South American developing countries, such as Uruguay (26.4%), Bolivia (40.5%), Colombia (31.7%), Paraguay (33.0%), and Argentina (41.0%)^11^. On the other hand, the prevalence of regular tobacco use (6.7%) was lower than the one found in students from other countries, such as the USA (12.7%)^1^, Canada (14.4%)^10^, Uruguay (9.2%), Colombia (12.5%), Bolivia (12.3%), Paraguay (14.7%), and Argentina (18.7%)^11^. In fact, the pattern of regular psychoactive substances use (particularly tobacco) varies substantially among countries in the American continent, reflecting the different methodological approaches, the magnitude of investigated samples and mainly the discrepancy in sociodemographic characteristics. In particular, the increase in age and male gender are the variables most responsible for disparities in rates of smoking among students. In addition to these factors, it is important to consider that the present study is prone to response bias and may have underestimated the results.

In Brazil, the prevalence of occasional tobacco use found here was higher than the multicenter study conducted in students attending PeNSE (33.4% *versus* 22.7%), however, the rate of regular use was similar between both types of research (6.7% *versus* 7.1%)^2^. It should be noted that the differences between the studies in regards to methodologies, the number of participants and other characteristics might have contributed to the disproportions between occasional smoking rates. PeNSE encompassed 61.037 ninth-grade students enrolled in elementary education level public and private schools of all Brazilian State capitals, using a self-applied questionnaire^2^. This study, however, examined only students of public schools, junior high, high school and EJA in the State of Goiás’ countryside and using face-to-face interviews. Therefore, more research is necessary to evaluate the differences in behavior related to the initiation and consumption patterns of tobacco in students, especially considering regional differences^12^.

This study found a higher prevalence of occasional tobacco use in male students, ratifying the greater vulnerability of men to tobacco use when compared to women. Smoking is considered more acceptable among men and can be a symbol of power, contributing to the disparity between the rates of tobacco use among genders^13^. Despite this finding, the consumption of tobacco by women has increased, indicating a higher risk of nicotine dependence and the development of tobacco-related diseases in females and epidemiological changes trend in the future^3^, driven by increased acceptance of this behavior in women because of the social rise of the female gender^6^.

In this study, we observed a positive gradient of occasional and regular tobacco consumption with aging, corroborating other investigations^2,3^. The systematic review pointed out that the use of tobacco is high in their early teens and still increases with advancing age^3^, which may reflect the increase of external influences (access to advertisements, parental and colleague influence) throughout life.

In this research, students who were children of smokers had a higher prevalence of occasional and regular tobacco use. In fact, prolonged exposure to parental smoking is associated with early experimentation and habitual consumption of tobacco by teenagers and young adults^14^. Systematic review ratifies that the use of tobacco by family members, especially parents, has a strong influence on the smoking behavior and an impact on increased nicotine dependence in adolescents^15^. Some mechanisms, such as positive attitudes about tobacco and availability of cigarettes in family environments, as well as imitation and learning this behavior from parents, are responsible for this intersection^16,17^.

Students with no religion presented a higher prevalence of regular tobacco consumption, as well as in other investigations. Religious belief is considered a strong protective factor against the use of psychoactive substances, because some religions impose more limits or even prohibit certain behaviors and conduct, including the use of psychoactive substances. In addition, religion can be a positive influence on the family structure, as well as in other lifestyles, and may contribute to decreasing the rates of tobacco use^18^.

This study found an association between family dysfunction and occasional and regular tobacco use, using the family APGAR instrument as an indicator. A study conducted in public university students in Colombia showed that having dysfunctional families is directly associated with the use of tobacco^19^. In India, an investigation conducted with 526 between 15 and 19 years of age found a strong association between family conflicts and teenage smoking^20^. Similarly, a study conducted with 4,786 adolescents and their parents in a mental health center in the United States showed that family involvement with the children’s daily activities is a protective factor against external problems that may lead to the initiation of tobacco use and the use of other psychoactive substances, while non-involvement contributes to early drug experimentation^7^.

Teenagers from dysfunctional families can be encouraged to use tobacco within the family environment or in other social spaces, such as in schools. This influence increases if these individuals are exposed to other risk factors, such as parental smoking, divorced parents or low-income families^21^. The family institution can play an important protective role, as well as be responsible for the exposure of adolescents to drugs, including alcohol and tobacco. Positive family influence requires paying attention to the teenager’s routine with adequate parental control and authority and family attention to coping situations for smoking and to the search for abstinence and help in cases of relapse^22^.

Among the students who smoked regularly, most presented nicotine dependence (74.5%). This result reinforces the need for health promotion and disease prevention among students, with emphasis on the effective practice of primary health care in and out of schools that include tracking the nicotine dependence in this population^6^.

Some limitations of this study must be considered when interpreting the results. The transversal nature of the investigation prohibits the establishment of a cause-and-effect relationship between the use of tobacco and the variables investigated. The data were self-reported, and may under or overestimate the results. In an attempt to ease such restrictions, we assured the confidentiality of this study during data collection and everything took place in a private environment, so the answers could be as reliable as possible.

The findings of this study indicated the high prevalence of occasional and regular tobacco use by students and its association with sociodemographic variables and family dysfunction. We must consider such factors in the practice of health promotion and disease prevention in the context of tobacco control programs and school health, looking to extend control actions in school environments, as well as to implement public policies that contemplate the intersection between families, community, and the healthcare sector. It is necessary to rethink strategies for intersectional planning, especially in education and health, increasing the surveillance of tobacco use by students and families. Finally, the results of this study show the importance of adopting APGAR in an intensive approach to individuals at risk for tobacco use, and confirms the use of FT in smoker care and in the development of future epidemiological studies and clinical trials.

## Footnotes

a World Health Organization. Global status report on noncommunicable diseases, 2010. Geneva; 2011 [cited 2015 Oct 28]. Available from: http://www.who.int/nmh/publications/ncd_report_full_en.pdf

b World Health Organization. Who Report on the Global Tobacco Epidemic, 2015: raising taxes on tobacco. Geneva; 2015 [cited 2015 Oct 7]. Available from: http://apps.who.int/iris/ bitstream/10665/178574/1/ 9789240694606_eng.pdf

c World Health Organization. Global status report on noncommunicable diseases, 2010. Geneva; 2011 [cited 2015 Oct 28]. Available from: http://www.who.int/nmh/publications/ncd_report_full_en.pdf

d World Health Organization (WHO). HIV and adolescents: guidance for HIV testing and counselling and care for adolescents living with HIV: recommendations for a public health approach and considerations for policy-makers and managers. Geneva; 2013 [cited 2015 Feb 28]. Available from: http://www.youngpeopleandhiv.org/files/HIV_Testing_guideline.pdf

e Instituto Nacional de Câncer. Vigilância de tabagismo em escolares: dados e fatos de 12 capitais brasileiras (VIGESCOLA). Rio de Janeiro; 2004 [cited 2015 Feb 28]. Available from: http://www.inca.gov.br/tabagismo/31maio2004/vigescola.pdf

f Pan American Health Organization. Latino families and youth: a compendium of assessment tools. Washington (DC); 2013 [cited 2015 Feb 28]. Available from: http://www.paho.org/hq/index.php?option=com_docman&task=doc_view&gid=23171&Itemid
